# UDP-Glucosyltransferases Induced by *Nosema bombycis* Provide Resistance to Microsporidia in Silkworm (*Bombyx mori*)

**DOI:** 10.3390/insects12090799

**Published:** 2021-09-07

**Authors:** Bin Yu, Qiuhua Yang, Junhong Wei, Guoqing Pan, Chunfeng Li, Zeyang Zhou

**Affiliations:** 1 State Key Laboratory of Silkworm Genome Biology, Southwest University, Chongqing 400715, China; yubin5868@outlook.com (B.Y.); YQH17320362547@163.com (Q.Y.); weijunhong@swu.edu.cn (J.W.); gqpan@swu.edu.cn (G.P.); 2 Chongqing Key Laboratory of Microsporidia Infection and Control, Southwest University, Chongqing 400715, China; 3 College of Life Sciences, Chongqing Normal University, Chongqing 401331, China

**Keywords:** *Bombyx mori*, innate immunity, UDP-glucosyltransferase, induced expression

## Abstract

**Simple Summary:**

*Nosema bombycis* (*N. bombycis*), an obligate intracellular eukaryotic parasite, is a virulent pathogen of the silkworm, that causes major economic losses. Although many studies have reported on *B. mori* host response to this pathogen, little is known about which genes are induced by *N. bombycis*. Our results showed that two *B. mori* uridine diphosphate-glucosyltransferases (UGTs) (BmUGT10295 and BmUGT8453) could be activated by *N. bombycis* and provide resistance to the microsporidia in silkworms. These results will contribute to our understanding of host stress reaction to pathogens and the two pathogen-induced resistant genes will provide a target for promoting pathogen resistance.

**Abstract:**

As a silkworm pathogen, the microsporidian *N. bombycis* can be transovarially transmitted from parent to offspring and seriously impedes sericulture industry development. Previous studies found that Uridine diphosphate (UDP)-glycosyltransferases (UGTs) are involved in regulating diverse cellular processes, such as detoxification, pigmentation, and odorant sensing. Our results showed that *BmUGT10295* and *BmUGT8453* genes were specifically induced in infected silkworms, but other BmUGTs were not. Tissue distribution analysis of the two BmUGTs showed that the transcriptions of the two BmUGTs were mainly activated in the midgut and Malpighian tubule of infected silkworms. Furthermore, there were significantly fewer microsporidia in over-expressed BmUGTs compared with the control, but there were significantly more microsporidia in RNA interference BmUGTs compared with the control. These findings indicate that the two BmUGTs were induced by *N. bombycis* and provided resistance to the microsporidia.

## 1. Introduction

Microsporidia are a group of obligate intracellular parasites that can infect nearly all animal phyla in nature [1,2]. Microsporidia infection can cause major economic losses; for example, *Nosema ceranae* causes serious disease in adult honey bees [3,4], *Enterocytozoon hepatopenaei* affects cultured shrimp [5,6], and *Cryptosporidium baileyi* impacts the digestive and/or respiratory tract of many bird species across various orders [7,8]. Moreover, infection with some microsporidia is a serious threat to human life and health [9,10,11]. Although there are many studies on microsporidia, there has not been sufficient research on host response because of the complexity of host–pathogen interactions and the differences among species [12,13,14]. The microsporidian *N. bombycis* was identified from the silkworm, *Bombyx mori*, in which it causes pebrine and leads to enormous economic losses in the silkworm industry [15]. Upon infection, innate immune responses of silkworms against the pathogen *N. bombycis* are activated, such as hemocytin [16] and *Bombyx* Turtle protein expression [17]. Moreover, the autophagy pathway is induced by *N. bombycis* infection [18,19], and apoptosis and reactive oxygen species production also change upon infection with *N. bombycis* [20].

Uridine diphosphate (UDP)-glycosyltransferases (UGTs) are found in all living organisms, including animals, plants, bacteria, and viruses. As a phase II enzyme in the detoxification system, UGTs catalyze the addition of sugars to a broad range of lipophilic molecules; this turns the lipophilic molecules into more water-soluble metabolites that can be easily excreted to regulate internal molecules and protect the cellular system from damage by toxic foreign compounds [21]. In insects, UGTs play a vital role in the biotransformation of exogenous and endogenous compounds from being hydrophobic to hydrophilic, which results in more efficient internal molecule regulation and excretion that prevents retention of toxic foreign compounds. For example, UGTs are involved in the detoxification of xenobiotics produced by the plants upon which they feed [22,23].

The UGTs that participate in detoxification of plant toxins can also result in cross-resistance to various pesticides [24,25,26,27,28], and therefore could be a crucial knock down target when developing novel pest control strategies to improve the natural toxicity of plants or chemicals to pests [29]. Besides being detoxification enzymes, UGTs are also involved in physiological processes [30,31,32], pigmentation [33], and odorant sensing [34,35]. In *B. mori*, many UGTs have been identified [36,37]; BmUGT10286 catalyzes quercetin 5-O-glucoside formation, which protects pre-pupae from the harmful effects of UV radiation during metamorphosis and facilitates green cocoon formation [38]. The green cocoon of silkworms that results from quercetin 5-O-glucosyltransferase is an evolved response to dietary toxins [39]. Moreover, BmUGT1 shows activity with flavonoids, coumarins, terpenoids, and simple phenols, which supports a role of this enzyme in detoxication processes [40], and BmUGT013829, which is highly expressed in larval and adult antennae, may be involved in insect olfaction [36]. Though UGTs are involved in detoxification, pigmentation, odorant sensing and cocoon formation, there are no any reports about the involvement of UGT in microbial stress response in insects.

*B. mori* is an economically important insect and a lepidopteran model for investigating gene functions. In previous studies, many genes of silkworm were found to be induced by *N. bombycis* that were involved in many signal transduction pathways [19,41,42,43,44]. However, there was no report that UGT was induced in silkworm, and there was also few reports that UGT was involved in the process of biological stress. In this study, we determined that *BmUGT10295* and *BmUGT8453* genes were induced by *N. bombycis* and provided resistance to microsporidia proliferation. Our study is the first report to find that UGT facilitate resistance to pathogens in insects.

## 2. Materials and Methods

### 2.1. Insect Rearing and Cell Lines

The *B. mori* strain Dazao was reared on an artificial diet (Nihonnosanko, Yokohama, Japan) and maintained at 25 °C under a photoperiod of 12 h light and 12 h dark.

BmN-SWU1, a *B. mori* cell line, was cultured in TC-100 medium (United States Biological Inc., Swampscott, MA, USA) with 10% fetal bovine serum and 1% penicillin/streptomycin (Gibco, Waltham, MA, USA) and maintained at 28 °C [45].

### 2.2. Immune Challenge

*N. bombycis* CQ1 (No. CVCC102059) was conserved in the China Veterinary Culture Collection Center. The mature spores of *N. bombycis* were purified from infected silkworms and stored in sterilized distilled water at 4 °C for later use.

The artificial silkworm diet was cut into small pieces, adding 10^6^ *N. bombycis* spores to each piece. Each silkworm in day 1 fifth instar was fed a small piece of artificial diet with *N. bombycis*. The silkworms that did not eat the diet within 20 min were eliminated. Control silkworms were orally administered with sterilized water. Finally, 200 silkworms were separately screened out and separately reared together in the infected and control groups. Three groups (six silkworms per group) were randomly selected at 3, 6, 12, 24, 48, and 72 h after oral challenge, and all tissues were harvested. The collected samples were stored at −80 °C and later used for total RNA extraction or protein extraction.

### 2.3. RNA Isolation, cDNA Synthesis, and Reverse Transcription Polymerase Chain Reaction (RT-PCR)

Total RNA isolation and cDNA synthesis were conducted as described in a previous study [46]. To investigate the transcription of BmUGT genes in *B. mori*, RT-PCR was performed using ExTaq (Takara, Tokyo, Japan). The PCR program using touchdown-PCR amplification was conducted under the following conditions: initial denaturation at 94 °C for 5 min; followed by 20 cycles of 94 °C for 40 s, annealing temperatures starting at 62 °C for 40 s (decreasing 0.5 °C/cycle), and 72 °C for 1.5 min; 30 cycles of 94 °C for 1 min, 55 °C for 40 s, 72 °C for 2 min; and a final extension at 72 °C for 10 min.

For RT-PCR, all BmUGT gene sequences were obtained from the InsectBase database (http://www.insect-genome.com/ (accessed on 20 March 2017)) and the KAIKObase database (http://sgp.dna.affrc.go.jp/KAIKObase/ (accessed on 20 March 2017)). All gene IDs are listed in Table S1. The silkworm actin3 (BmA3) gene was used as an internal control and all primer sequences are listed in Table S2. The PCR product was analyzed by 1% agarose gel electrophoresis.

### 2.4. Gene Cloning

To obtain the full-length cDNA of BmUGT10295, we used a GeneRacer™ Kit (L1502, Invitrogen, Waltham, MA, USA) to synthesize the cDNA following the kit’s instructions. The primers used are listed in Table S2. PCRs were performed under the following conditions: 94 °C for 3 min; 30 cycles of 94 °C for 30 s, 55 °C for 30 s, and 72 °C for 1 min; and a final extension at 72 °C for 10 min. The purified PCR products were inserted into the PESI-Blunt simple vector (10910, Yeasen, Shanghai, China) and the positive clones were sequenced by the Sangon Company (Shanghai, China).

To obtain the 5′-end sequences of BmUGT10295 according to predicted transcription initiation sites, we designed the forward primers (BmUGT10295-TSS-F; Table S2). The 5′-end sequences of BmUGT10295 were obtained by PCR using the primers BmUGT10295-TSS-F and Bm10295-race-R (Table S2). The positive bands of PCR were cut and inserted into the PESI-Blunt simple vector. Then the positive clones were sequenced by the Sangon Company.

Because the sequence of the BmUGT8453 gene in KAIKObase was full-length cDNA, the *BmUGT8453* gene was also cloned and sequenced for verification. The primers used are listed in Table S2.

### 2.5. Real-Time Quantitative PCR (qPCR) Analysis

SW22934, a microarray probe of *Bombyx mori* eukaryotic transcription initiation factor 4A, was used as an internal control for normalization. The 20-μL mixture included 2 μL cDNA or DNA, 0.5 μL of each primer (10 mM; Table S2), 10 μL SYBR Green Master Mix reagent (Yeasen, Shanghai, China), and 7 μL ddH_2_O. qPCR was performed according to the following parameters: one cycle of an initial denaturation step at 95 °C for 5 min, followed by 40 cycles at 95 °C for 10 s, 60 °C for 20 s, and 72 °C for 40 s. The relative gene expression levels were estimated according to the 2^−ΔΔCt^ or 2^−ΔCt^ method [45]. These experiments were repeated three times and all samples were run in triplicate of each time.

### 2.6. Vector Constructs

Because there were substantial sequence similarities between *BmUGT10295* and *BmUGT8453* (Figure S2, a partial *BmUGT8453* sequence and the full-length *BmUGT10295* sequence were cloned into pET32a vector to generate pET32-BmUGT10295 and pET32-BmUGT8453 vectors for polyclonal antibody production. The primers are listed in Table S2.

For overexpression of the BmUGTs in BmN-SWU1 cell line, the pEHI vector was constructed. The region of the *B. mori* nuclear polyhedrosis virus (BmNPV) genome that contained homologous region 3 (HR3), which acts as an enhancer for the promoter of a nonviral gene, were cloned from BmNPV. The OpIE2-MCS-PA fragment (OpIE2 promoter, multiple cloning sites, and polyadenylation sequence) was cloned from the pIZ/V5-His vector (Invitrogen, Waltham, MA, USA). The HR3-OpIE2-MCS-PA fragment was assembled with the fragment HR3 and OpIE2-MCS-PA using overlapping PCR techniques. Then, the HR3-OpIE2-MCS-PA fragment was inserted into the PESI-Blunt simple vector (10910, Yeasen, Shanghai, China) to obtain the pEHI vector. The full-length *BmUGT10295* and *BmUGT8453* were cloned from the above PESI vectors. *DsRed* was cloned from the pDsRed2-N1 vector. Then full-length *BmUGT10295*, *BmUGT8453* and *DsRed* sequences were cloned into the pEHI vector to generate pEHI-BmUGT10295, pEHI-BmUGT8453 and pEHI-DsRed vectors. The primers are listed in Table S2.

### 2.7. Protein Expression, Purification, and Polyclonal Antibody Production

The above pET32a recombinant plasmids (pET32-BmUGT10295 and pET32-BmUGT8453 vector) were transformed into BL21(DE3) cells for BmUGT expression following standard protein expression protocols. Briefly, when the culture reached an OD_600_ of 0.4–0.6, it was induced with 0.1 mM isopropyl-b-D-1-thiogalactopyranoside for 20 h. The cells that contained recombinant vector were re-suspended in lysis buffer (20 mM Tris-HCl, pH 8.0, and 100 mM NaCl) and sonicated. Then, the fused expression proteins were purified using the Ni–NTA beads (QIAGEN, Valencia, CA, USA).

For polyclonal antibody production, all animal experiments were conducted in accordance with Laboratory Animals Ethics Review Committee of Southwest University guidelines (Chongqing, China), and the committee approved this study (Permit Number: AERCSWU2017-7). Three mice were each subcutaneously inoculated with each recombinant BmUGT10295 and BmUGT8453 (80–120 μg/mouse) homogenized with Freund’s adjuvant (1:1; Sigma, St. Louis, MO, USA) four times. One week after the fourth injection, antisera were collected and stored at −80 °C.

### 2.8. Western Blotting

The silkworm samples were ground with liquid nitrogen and then lysed with RIPA (P0013B, Beyotime Biotechnology, Shanghai, China) at 4 °C for 30 min. The supernatants were separated by SDS–PAGE and transferred to a PVDF membrane (Roche, Basel, Switzerland). After blocking for 1 h at 37 °C in TBST (20 mM Tris-HCl, 150 mM NaCl, 0.05% Tween-20) with 5% (*w*/*v*) skim milk, membranes were incubated with 1:1000 dilutions of anti-BmUGT or negative control serum in TBST for 1 h at 37 °C. Following several washes, membranes were reacted with HRP-labeled goat anti-mouse IgG (Bio-Rad, Richmond, CA, USA), successively, with washing in between. ECL Plus Western Blotting Detection Reagents (Bio-Rad, Richmond, CA, USA) were used to detect the bound antibodies.

### 2.9. Indirect Immunofluorescence Assay 

To detect BmUGT expression, we plated the *B. mori* cell line BmN-SWU1 in 6-well culture plates (10^5^ cells/well) and challenged with *N. bombycis* (spore: cell, 10:1). Then, 72 h after infection, infected cells were fixed in 4% paraformaldehyde and permeabilized with 0.5% Triton X-100 for 5 min. The cells were subsequently blocked in PBS that contained 10% (*w*/*v*) goat serum and 0.5% (*v*/*v*) BSA for 1 h and incubated with anti-BmUGT (1:1000) for 1 h. Alexa488 was used to label the primary antibodies and DNA was stained with DAPI (Sigma, St. Louis, MO, USA) for 30 min. Fluorescence was observed and imaged with confocal microscopy (Olympus, Tokyo, Japan).

### 2.10. dsRNA Synthesis

The interference segments of BmUGT10295, BmUGT8453, and EGFP (control) were designed by the siDirect database (http://sidirect2.rnai.jp/ (accessed on 30 May 2018)). T7 promoter sequences were tailed to sense and antisense primers (primers sequences in Table S2). dsRNA synthesis was conducted with a Transcript Aid T7 High Yield Transcription Kit (KO441, Thermo Scientific, Waltham, MA, USA), which were purified using a MicroElute RNA Clean-up Kit (R6247, OMEGA, Doraville, GA, USA) for RNA interference (RNAi).

### 2.11. Overexpression and RNAi BmUGTs

For overexpression of *BmUGT10295* and *BmUGT8453*, BmN-SWU1 cells were transiently transfected with pEHI-BmUGT10295 or pEHI-BmUGT8453 expression plasmids (3 µg), and pEHI-DsRed (3 µg) was used as a control. Then, these cells were challenged with *N. bombycis* (spore: cell, 10:1).

For RNAi *BmUGT10295* and *BmUGT8453*, BmN-SWU1 cells were transiently transfected with dsRNA of *BmUGT10295* or *BmUGT8453* (3 µg), and the dsRNA of EGFP was used as a control. Then, these cells were challenged with *N. bombycis* (spore: cell, 10:1).

Each of the above cells was harvested on 1, 3 and 5 days post-infection *N. bombycis*; They were divided into two parts and broken with glass beads. One part was used for RNA extraction (R6934, OMEGA, Doraville, GA, USA) and the other was used for DNA extraction (D3396, OMEGA, Doraville, GA, USA). The cDNA was synthesized to estimate the effects of overexpression and RNAi BmUGTs through RT-qPCR (reverse transcription–qPCR.). The DNA was used to estimate the relative copy levels of *N. bombycis* infection through qPCR.

### 2.12. Statistical Analysis

One representative data set of three experiments was used to generate figures with GraphPad Prism 8. All statistical analyses were conducted using IBM SPSS v. 22. All results are shown as means ± SD of triplicate samples. All data presented are representative of a minimum of three independent experiments.

## 3. Results

### 3.1. Identification of the N. bombycis-Inducible BmUGT Genes

#### 3.1.1. Transcription of BmUGT Genes in *B. mori*

**Figure 1:**
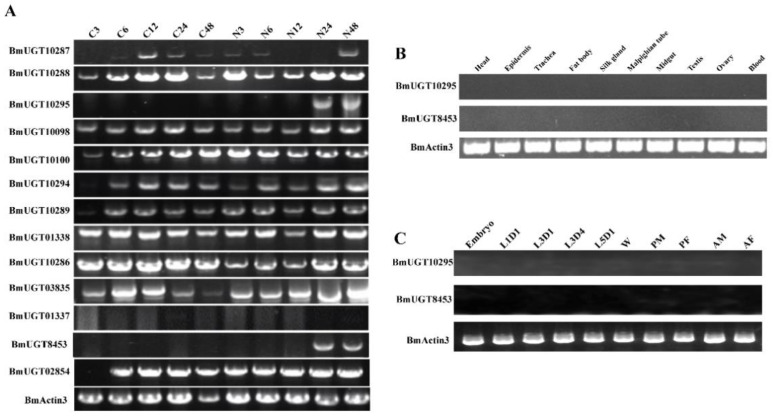
Transcription of *Nosema bombycis*-inducible BmUGT genes. (**A**): Different expression patterns of BmUGTs in response to *N. bombycis*. N3–N48 represents the silkworm samples at 3~48-h post-infection, whereas C3–C48 represent the control group, which was given water. (**B**): *BmUGT10295* and *BmUGT8453* transcription in the different tissues of uninfected silkworms on day 3 fifth instar larvae using actin3 (BmActin3) as internal reference. (**C**): *BmUGT10295* and *BmUGT8453* transcription in different larval growth stages. RT-PCR was used to analyze the expression characteristics of *BmUGT10295* and *BmUGT8453* in embryos, 1-day-old first instar larvae (L1D1) to 1-day-old fifth instar larvae (L5D1), wandering larvae (W), male and female pupae (PM/PF), and male and female adults (AM/AF) of uninfected *B. mori*.

In our previous transcriptome data induced by *N. bombycis* (unpublished data), the transcription of *BmUGT10295* was activated in infected silkworms. In previous research, 42 UGT genes were identified in *B. mori*, which is much more than that known in other insects, and they belonged to five groups that were identified by phylogenetic analysis. The *BmUGT10295* gene belongs to the Group I cluster, which is silkworm-specific [36]. To investigate whether the other *B. mori* UGT genes were induced by *N. bombycis* in silkworms, all genes of the Group I cluster were analyzed in infected and uninfected silkworms. The results showed that only the *BmUGT10295* and *BmUGT8453* genes activated transcription in infected silkworms (Figure 1A).

The transcriptional activation of *BmUGT10295* and *BmUGT8453* genes in different silkworm tissues showed that there was no transcription in any tissues of uninfected silkworm (Figure 1B). There was also no transcriptional activation in any stages of uninfected silkworms (Figure 1C). These findings indicate that *BmUGT10295* and *BmUGT8453* genes are the only UGT genes activated by *N. bombycis* [36].

#### 3.1.2. Transcription of BmUGT10295 and BmUGT8453 Genes in Different *N. bombycis*-Infected *B. mori* Tissues

**Figure 2:**
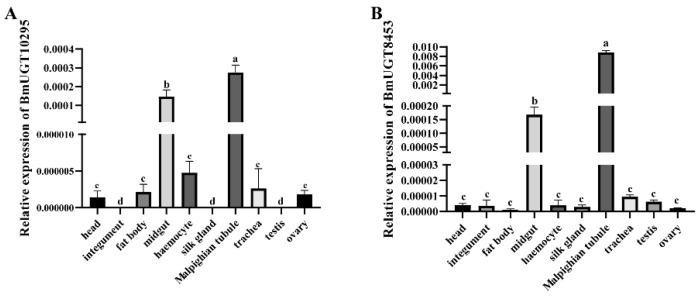
Spatial expression patterns of infected *B. mori*. Day 1 fifth instar *Bombyx mori* that were infected with *Nosema bombycis.* The relative expressions of *BmUGT10295* (**A**) and *BmUGT8453* (**B**) were assessed in different tissues of silkworms 3 days after infection. The relative gene expression levels were estimated according to the 2^−ΔCt^ method. sw22934 was used as an internal reference. Bars represent the mean of three individual measurements ± SD. Statistical analysis was conducted by one-way ANOVA using a Tukey’s multiple comparison test. Identical letters indicate no significant difference (*p* > 0.01), whereas different letters indicate a significant difference (*p* < 0.01).

Although the *BmUGT10295* and *BmUGT8453* genes were induced in infected silkworms, their transcription in different tissues during infection is unknown. Using *N. bombycis*-infected silkworms, the transcriptional levels of *B. mori BmUGT10295* and *BmUGT8453* genes were analyzed by RT-qPCR in different tissues at 48 h post-infection. The transcription of the *BmUGT10295* and *BmUGT8453* genes was detected, and mainly in the midgut and Malpighian tubule (Figure 2). According to the MIQE guidelines [47], the primer amplification efficiencies were also carried out. The results showed amplification efficiencies of BmUGT10295 and BmUGT8453 were 105% and 103% respectively, which is generally considered acceptable (Figure S1).

### 3.2. Full ORF Clone of the BmUGT Genes

**Figure 3:**
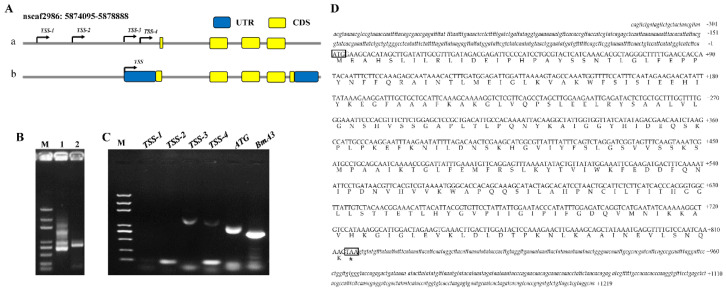
Obtaining the *BmUGT10295* full-length cDNA. (**A**): The schematic phase included structural information of *BmUGT10295* in the genome. (**a**), Annotation information of BmUGT10295 in genome; TSS-1~TSS-4 represented the predicted transcription initiation site; (**b**), Schematic diagram of *BmUGT10295* gene structural information corrected according to experimental results. The yellow squares represent coding DNA sequences (CDS), whereas the blue squares represent untranslated regions (UTR). (**B**): 3′ RACE of *BmUGT10295*; 1 is from the first round of PCR and 2 is from the nested PCR. (**C**): Determination of transcription start sites (TSSs). (**D**): The full-length cDNA sequence of *BmUGT10295*.

Although the *BmUGT10295* gene was identified, the sequence information it contains remains controversial [36,37]. The predicted DNA sequence of *BmUGT10295* was 780 bp and composed of four exons that encode 260 amino acids, as inferred in the InsectBase database (http://www.insect-genome.com/ (accessed on 20 March 2017)) (Figure 3Aa). According to this sequence, the 3′ end of *BmUGT10295* was cloned by 3′ RACE (Figure 3B) and the 3′ end sequence was acquired by sequencing. Unfortunately, 5′ RACE failed. Then, the transcription start site (TSS) of *BmUGT10295* was predicted (Figure 3Aa). Based on these predicted TSS sites, the primers were designed and PCR was performed. The PCR result showed that there were significant amplification bands in TSS-3, TSS-4 and ATG group (BmA3 was used as a control) (Figure 3C). Then, the PCR products were purified, cloned into a PESI vector, and sequenced. The clone and sequence results of TSS-3 were consistent with the genome sequence (Figure 3D). So, the transcriptional initiation of *BmUGT10295* was TSS-3. Therefore, the full cDNA of the *BmUGT10295* gene was obtained, which was 1550 bp and composed of five exons that encode 271 amino acids (Figure 3Ab,D). Moreover, multiple sequence alignment showed that *BmUGT10295*, *BmUGT8453*, and *BmUGT1* were highly conserved (Figure S2). Because the sequence of *BmUGT8453* gene in KAIKObase was full-length cDNA, the *BmUGT8453* gene was also cloned, which the sequencing result was coincident with KAIKObase data.

### 3.3. Recombinant BmUGT Purification and Immunoblot Analysis

**Figure 4:**
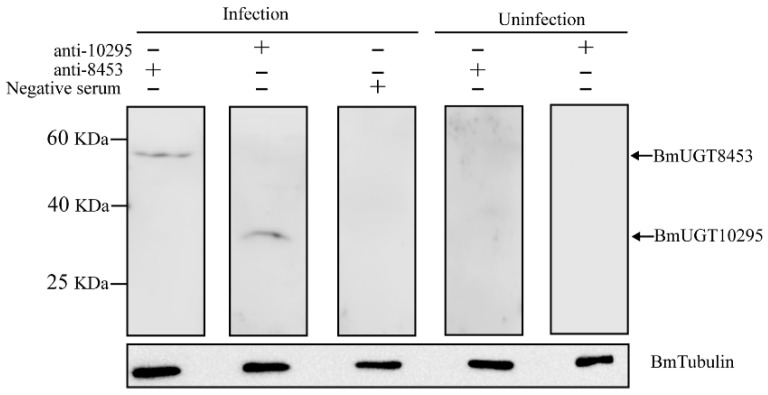
Western blotting analysis of BmUGT expression in infected silkworms. The polyclonal antibodies of BmUGT10295 (anti-10295) and BmUGT8453 (anti-8453) recognized the corresponding signal in infected silkworms, but not in uninfected silkworms. BmTubulin was used as an internal reference.

The above results showed that *BmUGT10295* and *BmUGT8453* transcription was detected, but their expression was unknown. Because the DNA sequences of *BmUGT10295* and *BmUGT8453* are very similar (Figure S2), the differential parts of *BmUGT10295* and *BmUGT8453* genes were successfully integrated into the pET-32a vector for protein expression in *E. coli.* SDS–PAGE analysis showed that recombinant BmUGTs (rBmUGTs) were expressed at a molecular mass of ∼40 kDa, which was consistent with the predicted size (Figure S3A,B). The purified target proteins were cut from the gel and used to prepare the antibody (Figure S3C). Western blot indicated that the rBmUGT antisera specifically recognized an approximately 40-kDa protein (Figure S3D). Then, the antisera of BmUGTs were used to detect the BmUGT10295 and BmUGT8453 expression. The results showed that there were only blot signals in infected silkworms. Expressed BmUGT8453 had a molecular mass of ∼60 kDa, which was consistent with the predicted size. Additionally, expressed BmUGT10295 had a molecular mass of ∼30 kDa, which was also consistent with its predicted size and indicated that the ORF was full-length (Figure 4).

### 3.4. Nosema Bombycis Inhibited by BmUGT10295 and BmUGT8453

**Figure 5:**
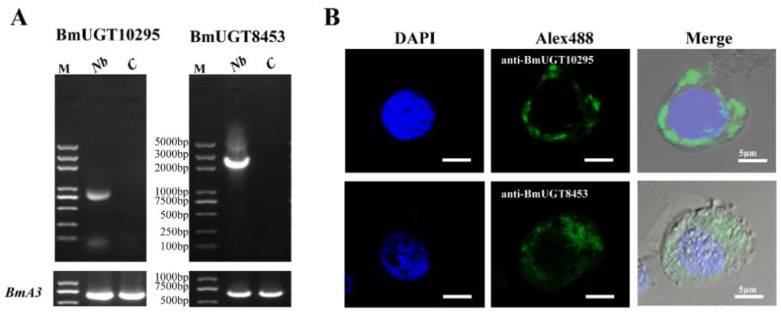
BmUGTs induced by *Nosema bombycis* in the infected BmN-SWU1 cell line. (**A**): RT-PCR showed that the BmUGT expression was activated by *N. bombycis* (Nb) in the infected cell line but not in control. *Bombyx mori* actin3 (BmA3) was the internal reference. (**B**): Localization of BmUGTs in the infected BmN-SWU1 cell line. Green fluorescence was observed in the samples incubated with the polyclonal antibodies of BmUGTs. Blue fluorescence represents nuclei labeled with DAPI (Sigma, Saint Louis, MO, USA).

Because *BmUGT10295* and *BmUGT8453* are induced by *N. bombycis*, more research was needed to elucidate the role of *BmUGT10295* and *BmUGT8453* in response to infection. Therefore, a cell-induced expression model was established in the BmN-SWU1 cell line. The results showed that *BmUGT10295* and *BmUGT8453* were only activated in infected cells (Figure 5).

**Figure 6:**
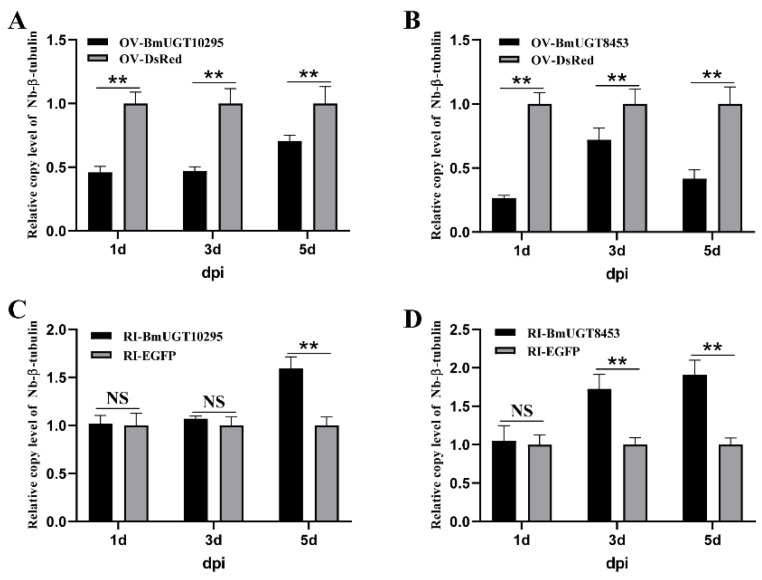
*BmUGT10295* and *BmUGT8453* inhibited *Nosema bombycis* infection. The *N. bombycis* β-tubulin relative copy levels in over-expressed BmUGT10295 (**A**) and BmUGT8453 (**B**) cells were lower than that in over-expressed DsRed cells. The *N. bombycis* β-tubulin relative copy levels in RNAi BmUGT10295 (**C**) and BmUGT8453 (**D**) cells were higher than that in RNAi EGFP cells. The relative copy levels were estimated according to the 2^−ΔΔCt^ method. sw22934 was used as an internal reference. Over-expressed DsRed and RNAi EGFP were used for calibration (value 1). Bars represent the mean of three individual measurements ± SD. Statistical significance was determined by an unpaired t-test, and statistically significant differences are represented with asterisks (** *p* < 0.01).

Furthermore, the amount of *N. bombycis* proliferation was evaluated in over-expressing BmUGTs cells. The results showed that the proliferation amount of *N. bombycis* was significantly lower in over-expressing BmUGT10295 and BmUGT8453 cells than in the control group (over-expressing DsRed cells) (Figure 6A,B). Additionally, the proliferation amount of *N. bombycis* in RNAi BmUGTs in BmN-SWU1 cells was much higher than in the control group (Figure 6C,D). The effects of over-expression and RNAi are shown in Figure S4.

## 4. Discussion

Silkworm, as a model of Lepidoptera, has significant economic and scientific values. *Nosema bombycis*, a pathogen that causes pebrine disease, can cause severe damage to the sericulture industry. However, few studies have examined the mechanism of host response to microsporidia infection. In previous studies, many genes were found to be induced by *N. bombycis* that were involved in many signal transduction pathways and are therefore key components of many cellular processes [19,41,42,43,44]. Although many genes were found to be activated by *N. bombycis* through a genome-wide survey in our previous study [41], these genes were determined to have different levels of background expression in subsequent work [48]. In addition, the Hsp70 promoter was reported to have relatively high microsporidia-inducible activity [49]. In this study, *BmUGT10295* and *BmUGT8453* were screened because they were activated by silkworm post-infection with *N. bombycis* but not in uninfected silkworms (Figure 1).

The sterile insect technique is a highly effective area-wide pest control tool with a low environmental impact, and is primarily used to control mosquitos [50,51,52] and flies [53]. There have been many sterile insect technique strategies, and one strategy is to use a sex-specific promoter or enhancer to drive the expression of a toxic gene for sex-specific death [54,55]. Microsporidia, an obligate intracellular parasite, must rely on cells to survive. Based on the SIT strategy, a toxic gene, driven by a *N. bombyx*-induced promoter, expressed explicitly in infected cells, could finally lead to cell death and microsporidia lost its living host. In this way, the silkworm was provided engineer resistance to *N. bombycis*. A previous study showed that an Hsp70 promoter-inducible genome editing system induced resistance to *N. bombycis* in transgenic silkworms [49], which picked up similar threads. Numerous genes can be up-regulated after microsporidia infection [41,42,56], but few specific microsporidia-inducible genes or promoters were reported. Though most UGTs are constitutively expressed in organisms, in this study, *BmUGT10295* and *BmUGT8453* were induced after microsporidia infection but had hardly any transcriptional activity in uninfected silkworms (Figure 1). Therefore, the research in this study provides a novel and effective target for promoting silkworm resistance to *N. bombycis*.

Members of the UGT superfamily typically catalyze the reaction of the covalent addition of sugar from UDP-sugar cofactors to a lipophilic acceptor, which is a second-order nucleophilic substitution reaction. UGTs can be divided into two major functional domains [57]. The N-terminal domain, which is variable in sequence between different isoforms, is responsible for binding the aglycone. Alternatively, the C-terminal domain, which is more conserved in sequence, is believed to contain a binding site for the UDP-sugar. For insects, UGTs prominently detoxify xenobiotic compounds from the plant on which they feed. For example, *Spodoptera frugiperda* uses SfUGT33F28 to inactivate maize defensive benzoxazinoids [58]. Moreover, nicotine, one of the most abundant secondary plant metabolites in tobacco, is highly toxic to herbivorous insects. In *Myzus persicae nicotianae*, UGTs could be required to detoxify nicotine [22]. In addition to plant xenobiotic tolerance, insect UGTs are also involved in insecticide detoxification. For example, the UGTs of *Aphis gossypii* are involved in sulfoxaflor [59,60], spirotetramat [24], imidacloprid [61], and other insecticides [62] resistance. four *Spodoptera* UGT genes are significantly co-up-regulated by the lambda-cyhalothrin, chlorantraniliprole, metaflumizone, and indoxacarb insecticides [27]. UGT201D3 is highly expressed and more inducible with abamectin exposure in the abamectin-resistant *Tetranychus cinnabarinus* strain [63]. Besides response to insecticides, UGT genes are also a response to pathogens. *Caenorhabditis elegans* UGT29 was robustly induced by *Burkholderia pseudomallei* [64]. Unlike *Caenorhabditis elegans,* there are no reports about the involvement of UGT in microbial stress response in insects. In this study, over-expressed or RNAi BmUGTs could affect the number of *N. bombycis* (Figure 6). Our study is the first report to find that UGT facilitates resistance to pathogens in insects and further analysis is required to reveal this mechanism.

## 5. Conclusions

*BmUGT10295* and *BmUGT8453* were activated by *N. bombycis* in infected silkworms. Moreover, these two BmUGTs provided resistance to microsporidia in the BmN-SWU1 cell line. The obtained results contribute to our understanding of host stress reaction to pathogens and provide a novel and effective target for promoting pathogen resistance.

## Data Availability

All data are available in the article.

## Supplementary Materials

The following are available online at https://www.mdpi.com/article/10.3390/insects12090799/s1, Figure S1: The amplification efficiency of primers in BmUGT10295 and BmUGT8453 gene; Figure S2: Multiple alignment of 3 BmUGTs; Figure S3: Purification of rBmUGTs and identification of BmUGTs polyclonal antibodies; Figure S4: The effect evaluations of over-expression and RNAi BmUGTs; Table S1: List of the detected BmUGT genes in *B. mori*; Table S2: Oligonucleotide primers.
