# Supplementary material for: UDP-Glucosyltransferases Induced by Nosema bombycis Provide Resistance to Microsporidia in Silkworm (Bombyx mori)
Source: Insects. 2021 Sep 7;12(9):799. doi: 10.3390/insects12090799 (PMC8469862; doi:10.3390/insects12090799)
Supplement: Supplementary file 1 [file insects-12-00799-s001.zip › insects-1346214-supplementary.pdf]

Supplemental Data

Figure S1

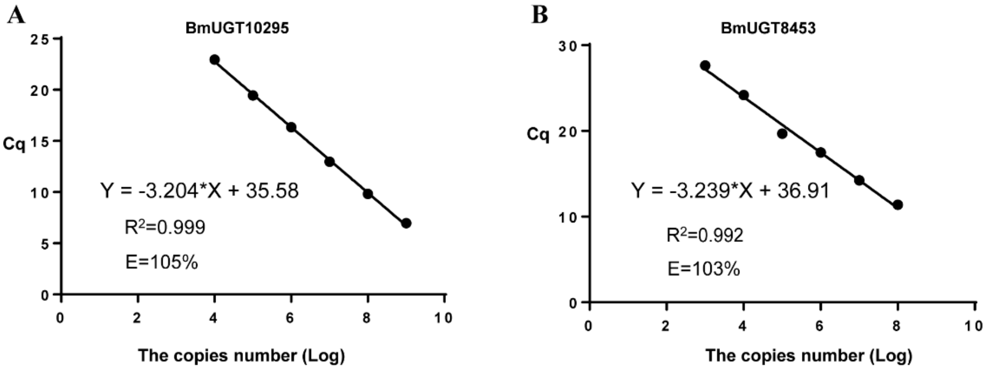

Figure S1. The amplification efficiency of primers in *BmUGT10295* and *BmUGT8453* gene. The standard curve was established to evaluate the amplification efficiency of primers. The templates with different copy numbers were pEHI-BmUGT10295 (A) and pEHI-BmUGT8453 (B) vectors respectively. All samples were run in triplicate. The figures and linear regression analysis were generated with GraphPad Prism 8. According to the MIQE guidelines, PCR efficiency (E) = $10^{-1/slope} - 1$.

Figure S2

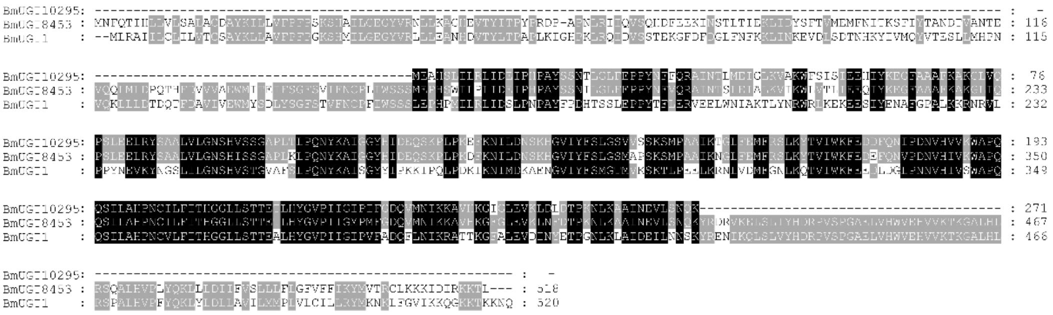

Figure S2. Multiple alignment of 3 BmUGTs. Multiple alignment was performed by using CLUSTAL in MEGA8.0 software. Amino acid sequence alignment between BmUGT10295 (KAIKObase accession number: KAIKOGA033056), BmUGT8453 (KAIKObase accession number: AK378453) and BmUGT1 (NCBI accession number: AF324465). Identical and similar residues are highlighted in black and grey, respectively.

Figure S3

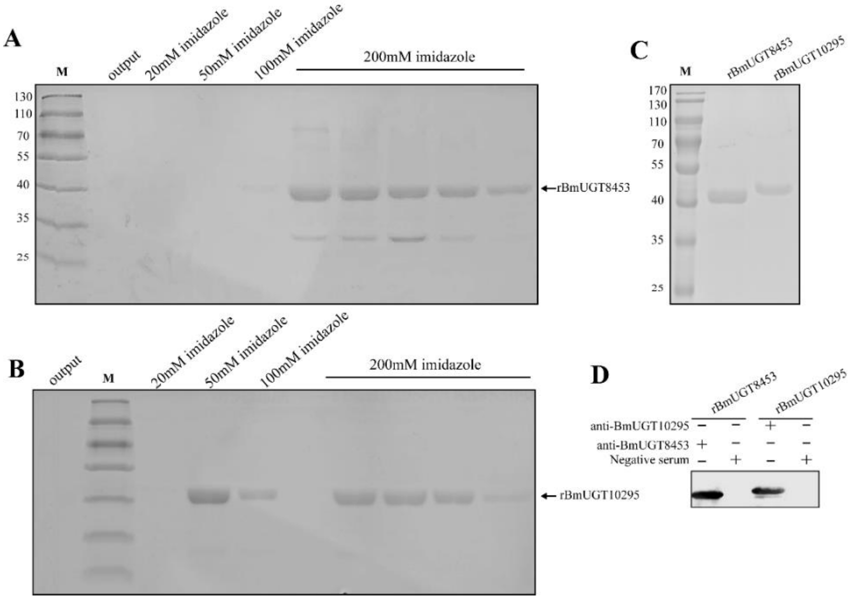

Figure S3. Purification of rBmUGTs and identification of BmUGTs polyclonal antibodies. Recombinant BmUGTs were eluted by elution buffer contained different concentration imidazole. The SDS-PAGE showed rBmUGT10295 (A) and rBmUGT8453 (B) was purified from recombinant *E. coli* by affinity chromatography, and the most of rBmUGTs were eluted in elution buffer which contained 200mM imidazole. The purified rBmUGTs were cut and detected (C) for inoculating mice. The polyclonal antibodies were produced in mice after injecting rBmUGT, and the polyclonal antibodies could specifically recognize rBmUGT (D).

Figure S4

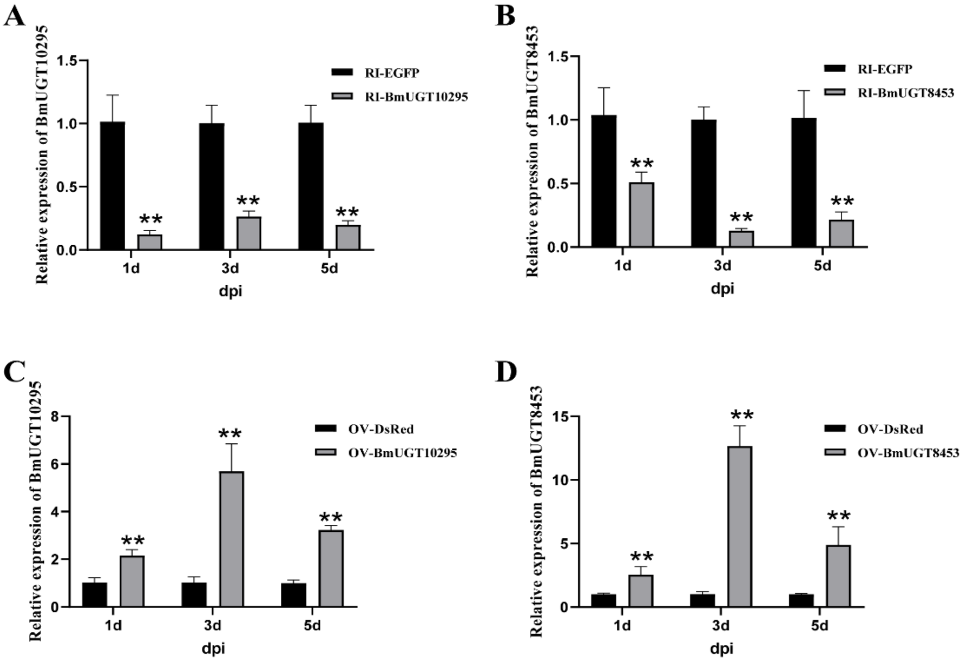

Figure S4. The effect evaluations of over-expression and RNAi BmUGTs. When interfering BmUGTs, the relative expression level of BmUGTs in RNAi BmUGT10295 cells (A) and in RNAi BmUGT8453 cells (B) was lower than that in RNAi EGFP cells. The relative expression level of

BmUGTs in over-expression BmUGT10295 cells (C) and over-expression BmUGT8453 cells (D) were higher than that in over-expression DsRed cells. The relative gene expression levels were estimated according to the $2^{-\Delta\Delta Ct}$ method. The sw22934 was used as internal reference. over-expression DsRed or RNAi EGFP were set as a calibrator (value 1). Bars represent the mean of three individual measurements ± SD. The statistical analysis was determined by an unpaired t-test and statistically significant differences are represented with asterisks (** $P < 0.01$).

Table S1. List of the detected BmUGT genes in *B. mori*

Gene Name	Gene ID[1]	Gene ID[2]	Size (Number of aa)
BmUGT10287	BGIBMGA010287	AB539965	431
BmUGT10288	BGIBMGA010288	AB539966	329
BmUGT10295	BGIBMGA010295	KAIKOGA033056	260
BmUGT10098	BGIBMGA010098	BMgn010098	316
BmUGT10100	BGIBMGA010100	AB539969	424
BmUGT10294	BGIBMGA010294	AF324465	319
BmUGT10289	BGIBMGA010289	AK377995	959
BmUGT01338	BGIBMGA001338	BMgn001338	365
BmUGT10286	BGIBMGA010286	AB539963	323
BmUGT03835	BGIBMGA003835	BMgn003835	422
BmUGT01337	BGIBMGA001337	BMgn001337	200
BmUGT8453	BGIBMGA010099	AK378453	518
BmUGT02854	BGIBMGA002854	AK377858	450

Gene ID[1], InsectBase database (http://www.insect-genome.com/); Gene ID[2], KAIKObase database (http://sgp.dna.affrc.go.jp/KAIKObase/);

Table S2. Oligonucleotide primers

Target gene and name	Sequence(5'→3')
The oligonucleotide primers for RT-PCR	
>BmUGT10287-F	GTAACGTATATAACCGCGTT
>BmUGT10287-R	ACAGTTTCTGGTACAAGGGT
>BmUGT10288-F	ACCCA AATCTCGAAT TCGAT
>BmUGT10288-R	CAGATTCTCGGTCCTGAGAT
>BmUGT10295-F	TTTCAGACTCCACGTTCACT
>BmUGT10295-R	TCCGACTTTGTGTAACTTCG
>BmUGT10098-F	AGCTTGGAGG GAATTGAGAT
>BmUGT10098-R	GGTACAAAGGCACGTGCAGT
>BmUGT10100-F	GCAAACGCTTTGATGACATT
>BmUGT10100-R	ACCGGACGATCATGGAAGAT
>BmUGT10294-F	CTCACGTGTCTACTGGAGTT
>BmUGT10294-R	CCACCCAGTGGACCAGCTCG
>BmUGT10289-F	GACTCACAATTGCAATAGCT
>BmUGT10289-R	ACCGGACGATCGTGATATAT

>BmUGT01338-F	ATGATGCCTCAGTTGGAAGC
>BmUGT01338-R	GGTCACGGAGGTGTAGAGGT
>BmUGT10286-F	AGTATTCGTCTCGTTGCATT
>BmUGT10286-R	AGGAGACACCGGACGATCGT
>BmUGT03835-F	ATGAGGTGTC TCGGACTGCTGTTCTT
>BmUGT03835-R	TCACCTATCATTGCTCAG
>BmUGT1337-F	ATGAGGTGTCTCGGACTGCTTTCTTCT
>BmUGT1337-R	CTACGCTACGTCCGGTCCG
>BmUGT8453-F	TTAAGTGCGCTCGCTTGCGA
>BmUGT8453-R	ATTGGTGCTCCGCTTGCGTT
>BmUGT2854-F	ATGAGGTGTC TCGGACTGCT
>BmUGT2854-R	TCACCTATCATTGCTCAG
>BmUGTA3-F	ATGGTGCGCTCCTCCAAGAACG
>BmUGTA3-R	CTACAGGAACAGGTGGTGGCGG

The oligonucleotide primers for full ORF clone cloning

>BmUGT10295-race-F	ATGGAGATTGGATTAAAAGTAG
>BmUGT10295-race-R	TTACTTTTGATTGGACAAAACC
>BmUGT10295-race-F2	TCCCACGTTTCTTCTGGAGC
>BmUGT10295-TSS-F1	TCCTTAAACTAAATATAAAG
>BmUGT10295-TSS-F2	ATCAAATCTAGTATCTGAGCTC
>BmUGT10295-TSS-F3	ACGAACAAAGTGTCACGTTC
>BmUGT10295-TSS-F4	GACCGAGATTTTATTTTAATTTG
>BmUGT8453-ORF-F	ATCATTCACAGTACGTATTAAG
>BmUGT8453-ORF-R	GAAACATTATTTATTGGTGC

The oligonucleotide primers for vector constructs

>Hr3-F-*Asc* I	*GGCGCGCC*CGGGAGATCTCAGCGTCGTGAAAAGAGGCAAT
>Hr3-R	ACCATACATTGTTTATCATCATGATCCATGGAAAAAGAAGCCGTGCCCAGT
>opIE2-F	ACTGGGCACGGCTTCTTTTTCCATGGATCATGATGATAAACAATGTATGGT
>opIE2-R-*Asc* I	*GGCGCGCC*AGATCTTGTTCTTTCCTGCGTTATCCCCT
>BmUGT10295-F-*Bam*H I-pEHI	AAGCTTGGTACCGAGCTC*GGATCC*ATGGAAGCACATAGCTTGAT
>BmUGT10295-R-*Xho* I-pEHI	ACCGCGGGCCCTCTAGA*CTCGAG*CGTTTTGATTGGACAAAACCT
>BmUGT8453-F-*Bam*H I-pEHI	AAGCTTGGTACCGAGCTC*GGATCC*ATGAATTTTCAAACGATTCA
>BmUGT8453-R-*Xho* I-pEHI	ACCGCGGGCCCTCTAGA*CTCGAG*CGAAAGTTTTTTTCTAATATCTAT
>DsRed-F-*Bam*H I-pEHI	AAGCTTGGTACCGAGCTC*GGATCC*ATGGTGCGCTCCTCCAAGAAC
>DsRed-R-*Xho* I-pEHI	ACCGCGGGCCCTCTAGA*CTCGAG*CGAGGAACAGGTGGTGGCGGCC
>BmUGT10295-F-*Bam*H I-pET32	*GGATCC*ATGGAAGCACATAGCTTGAT
>BmUGT10295-R-*Xho* I-pET32	*CTCGAG*TTTTTGATTGGACAAAACCT
>BmUGT8453-F-*Bam*H I-pET32	*GGATCC*ATGAATTTTCAAACGATTCA
>BmUGT8453-R-*Xho* I-pET32	*CTCGAG*TAACGCAATCTCCAACGAAGTG

The oligonucleotide primers for RT-qPCR

>BmUGT10295-qF	CTGGGTTGTGGGTACCAGAG
>BmUGT10295-qR	AGCTCAGGAAACACCTTGGG

>BmUGT8453-qF	TAAGTTTGCACCTCGCTGGT
>BmUGT8453-qR	CGCGTGTCAGCAACAAAGAT
>sw22934-qF	TTCGTACTGGCTCTTCTCGT
>sw22934-qR	CAAAGTTGATAGCAATTCCC
> Nb-β-tubulin-qF	AGAACCAGGAACAATGGAC
> Nb-β-tubulin-qR	AGCCCAATTATTACCAGCACC
The oligonucleotide primers for RNAi	
>RI-BmUGT10295-T7-F	TAATACGACTCACTATAGGGAGAGCAGTTCTGATAGTTCTGCT
>RI-BmUGT10295-T7-R	TAATACGACTCACTATAGGGAGAGCTCCATTGAAGATGACCATAT
>RI-BmUGT8453-T7-F	TAATACGACTCACTATAGGGAGATCATCCAAGTTTCACAGCAT
>RI-BmUGT8453-T7-R	TAATACGACTCACTATAGGGAGAGGCAGGATGGGGAATCTCGT
>RI-EGFP-T7-F	TAATACGACTCACTATAGGGAGAGGTGCCCATCCTGGTCGAGCT
>RI-EGFP-T7-R	TAATACGACTCACTATAGGGAGAGCTGGTAGTGGTCGGCGAGCT
